# Temporal and Spatial Regulation of *miR-320* in the Uterus during Embryo Implantation in the Rat

**DOI:** 10.3390/ijms11020719

**Published:** 2010-02-11

**Authors:** Hong-Fei Xia, Xiao-Hua Jin, Pei-Pei Song, Yi Cui, Chun-Mei Liu, Xu Ma

**Affiliations:** 1 Reproductive and Genetic Center, National Research Institute for Family Planning, Beijing 100081, China; E-Mails: hongfeixia@yahoo.com.cn (H.-F.X.); jxh969696@yahoo.com.cn (X.-H.J.); songqi_3587@163.com (P.-P.S.); cuiyi_6@hotmail.com (Y.C.); minnieliu@sohu.com (C.-M.L.); 2 Graduate School, Peking Union Medical College, Beijing 100005, China

**Keywords:** *miR-320*, embryo implantation, uterus, rat, hormone, pregnancy

## Abstract

The implantation process is complex, requiring reciprocal interactions between implantation-competent blastocysts and the receptive uterus. There were reports to show that some microRNAs (miRNAs) may play a key role during embryo implantation in mouse. However, the *miR-320* expression profiles in the rat uterus during peri-implantation are unknown. In the present study, we found that the expression level of *miR-320* was lower on day 5 of gestation (g.d. 5) in rats than g.d.3 and g.d.4 and restored gradually from g.d.6. *MiR-320* was specifically localized in glandular and luminal epithelia and decidua. The expression of *miR-320* was not significantly different in the pseudopregnant uterus and decreased in the uteri of rats subjected to activation of delayed implantation. Artificial decidualization and treatment with progesterone increased the *miR-320* expression. Thus, *miR-320* was differentially expressed in the rat uterus during implantation. The expression level was affected by active blastocysts and decidualization during the window of implantation. Steroid hormones, progesterone stimulated *miR-320* expression.

## 1. Introduction

Implantation is a highly coordinated sequence of events that begins with the attachment of an embryo to the uterine luminal epithelium and ultimately results in formation of the placenta. Implantation of the embryo to the uterine wall is regulated by various factors, for example, hormones [1,2], cytokines [3,4], growth factor [5,6], *etc*. Although a growing list of molecules are known to be involved in the implantation process, specifically, mechanisms associated with the onset of uterine receptivity and embryo implantation remains to be determined.

MicroRNAs (miRNAs) are small noncoding RNAs whose function as modulators of gene expression is crucial for proper control of cell growth [7,8] and are known to participate in mouse embryo implantation [9,10]. Mouse mir-320 was predicted [11] based on homology to a cloned human miRNA (MI0000542) [12] and cloned from mouse oocytes and testes [13]. The human *miR-320* sequence was originally cloned from the normal mucosa-derived population of human[12] and rat *miR-320* sequence was firstly found from large-scale cloning studies [14]. *MiR-320* expression level was down-regulated in primary breast cancer (BC) [15], and inhibited HL-60 cell proliferation by targets transferrin receptor 1 (CD71) [16]. This implied that *miR-320* may play an important role in the development of cancer. Embryo implantation shares similar phenomena and mechanisms with tumor invasion [17]. However, up to now, there is no information in the literature about how such miRNAs act on the rat uterus during embryo implantation.

Here, we report the expression pattern of *miR-320* in the uterus during embryo implantation in the rat. We studied the effect of pseudopregnancy, artificial decidualization and activation of delayed implantation on the expression of *miR-320*. In addition, we also tested the effect of steroid hormones on *miR-320* expression.

## 2. Results and Discussion

### 2.1. Differential expression of miR-320 in the rat uterus during the peri-implantation period

**Figure 1.**
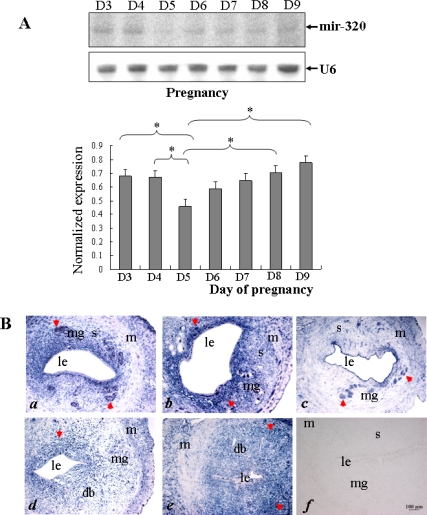
Changes in uterine *miR-320* expression during early pregnancy. (A) Northern blot analysis of uterine *miR-320* expression during days 3–9 of pregnancy. Hybridization was done with a ^32^P-labeled probe for *miR-320* and *U6*. The black curve represents the optical densities of the signals quantified by densitometric analysis and represented as a ratio of *miR-320* to *U6* intensity to normalize for gel loading and transfer. (B) *In situ* localization of *miR-320* in the rat uterus during early pregnancy. Sections of the uterus from days 3 (*a*), 4 (*b*), 5 (*c*), 6 (*d*), 7 (*e*) of pregnancy were subjected to *in situ* hybridization using DIG-labeled LNA probes specific to *mir-320*. Staining was developed using BCIP/NBT: blue staining indicates a hybridization signal. To evaluate the specificity of the probe, negative control staining was performed by substituting as DIG-labeled LNA-scrambled probe for the DIG-labeled LNA-miR-320 probe (*f*). The scale bar indicated a distance of 100 μM. Key: m, myometrium; mg, maternal gland; s, stroma; le, luminal epithelium; db, decidua basalis.

To study the role of *miR-320* in embryo implantation, we first examined its temporal and spatial distribution in the uterus during the peri-implantation period in rat. Northern blot analysis showed that the expression level of *miR-320* was lower on g.d. 5 in rats than g.d. 3 and g.d. 4 (p < 0.05), and restored gradually from g.d. 6, and was higher on g.d. 8 and g.d. 9 than g.d. 5 (p < 0.05) (Figure 1 A). The *in situ* hybridization results showed that the *miR-320* was mainly located in the glandular, luminal epithelia and stroma on g.d. 3 and g.d. 4 (Figure 1 B *a* and *b*). This suggested that it might participate in uterine epithelial and endometrial remodeling in preparing it to receive implanting blastocysts. On g.d. 5, the *miR-320* signal mainly appeared in the glandular epithelia and weak in the luminal epithelia and stroma (Figure 1 B *c*). A *miR-320* signal was found in the deciduas, glandular and luminal epithelia on g.d. 6 (Figure 1 B *d*) and was strengthened in deciduas from g.d. 7 (Figure 1 B *e*). In rat, blastocysts entered into uterus on g.d. 5 and began to implant from g.d. 6. In this study, the implantation sites and interimplantation sites in uterus were not examined independently and whole uterus was collected to perform the experiment of Northern blot and *in situ* hybridization after embryo implantation. The *in situ* results were from interimplantation uterine regions on g.d. 6. The above-mentioned observations suggested that the presence of blastocysts may reduce the expression of *miR-320* in the uterine luminal epithelia and stroma and decidualization may induce the expression of *miR-320* in pregnant rats.

### 2.2. Pseudopregnancy did not change the expression of miR-320

**Figure 2.**
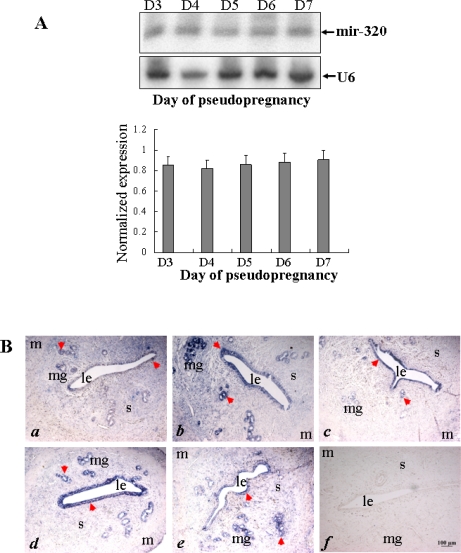
Changes in uterine *miR-320* expression during pseudopregnancy. (A) Northern blot analysis of uterine *miR-320* expression during days 3–7 of pseudopregnancy. Hybridization was done with a ^32^P-labeled probe for *miR-320* and *U6*. The black curve represents the optical densities of the signals quantified by densitometric analysis and represented as a ratio of *miR-320* to *U6* intensity to normalize for gel loading and transfer. (B) *In situ* localization of *miR-320* in the rat uterus during pseudopregnancy. Sections of the uterus from days 3 (*a*) 4 (*b*), 5 (*c*), 6 (*d*), 7 (*e*) of pseudopregnancy were subjected to *in situ* hybridization using DIG-labeled LNA probes specific to *mir-320*. Staining was developed using BCIP/NBT: blue staining indicates a hybridization signal. To evaluate the specificity of the probe, negative control staining was performed by substituting as DIG-labeled LNA-scrambled probe for the DIG-labeled LNA-miR-320 probe (*f*). The scale bar indicated a distance of 100 μM. Key: m, myometrium; m, maternal gland; s, stroma; le, luminal epithelium; db, decidua basalis.

To see whether the *miR-320* expression was dependent upon the presence of embryos, uterine tissues were subjected to Northern blot and *in situ* hybridization analysis (Figure 2 A). The expression level of *mir-320* detected by Northern blot was not significantly different in uterus during days 3–7 of pseudopregnancy. I*n situ* hybridization showed that the *miR-320* signal was mainly found in uterine glandular and luminal epithelia during days 3–7 of pseudopregnancy (Figure 2 B). The expression level of *miR-320* is higher during g.d.3–4 than g.d. 5–7 in the stroma. These results suggested that the *miR-320* expression is not dependent upon the presence of embryos in pregnant rats.

### 2.3. Delayed implantation inhibited the expression of miR-320

**Figure 3.**
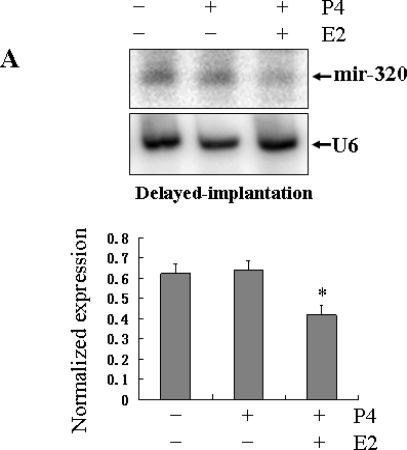

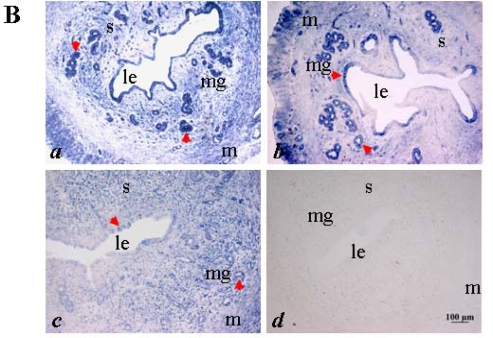
The expression of *miR-320* in the uterus of delayed implantation. (A) Northern blot analysis of uterine *miR-320* expression in a model of delayed implantation. Hybridization was done with a ^32^P-labeled probe for *miR-320* and *U6*. The black curve represents the optical densities of the signals quantified by densitometric analysis and represented as a ratio of *miR-320* to *U6* intensity to normalize for gel loading and transfer. (B) Sections of the uterus from delayed implantation (*a* and *b*) and activation of delayed implantation (*c*) were subjected to *in situ* hybridization using DIG-labeled LNA probes specific to *mir-320*. To evaluate the specificity of the probe, negative control staining was performed by substituting as DIG-labeled LNA-scrambled probe for the DIG-labeled LNA-miR-320 probe (*d*). The scale bar indicated a distance of 100 μM. Key: m, myometrium; m, maternal gland; s, stroma; le, luminal epithelium; db, decidua basalis.

To test whether the *miR-320* expression was dependent upon embryo implantation status, a delayed implantation model was used for Northern blot and *in situ* hybridization analyses. Northern blot showed a high level of *miR-320* in the uterus under delayed implantation conditions, but it decreased significantly after implantation was activated with estrogen treatment (*P* < 0.05; Figure 3 A). *In situ* hybridization showed that a strong signal appeared in the uterine glandular and luminal epithelia and weak in stroma during delayed implantation (Figure 3 B *a*, *b*). After implantation was activated by estrogen treatment and the embryos had implanted, there was weak *miR-320* expression in the stroma, glandular and luminal epithelia (Figure 3 B *c*), suggesting that down-regulation of *miR-320* expression was dependent upon the presence of viable implanting blastocysts.

### 2.4. Experimentally induced decidualization increases the expression of miR-320

**Figure 4.**
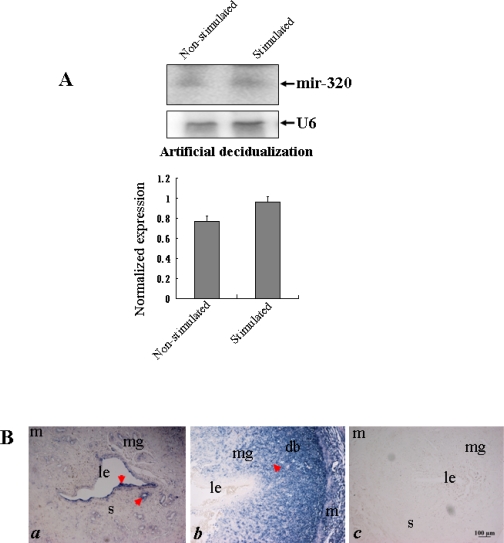
The expression of *miR-320* in the uterus of artificial decidualization. (A) Northern blot analysis of uterine *miR-320* expression in a model of artificial decidualization. Hybridization was done with a ^32^P-labeled probe for *miR-320* and *U6*. The black curve represents the optical densities of the signals quantified by densitometric analysis and represented as a ratio of *miR-320* to *U6* intensity to normalize for gel loading and transfer. (B) *In situ* localization of *miR-320* in the uterus of rats showing artificial decidualization. Artificial decidualization was stimulated by infusing 100 μL sesame oil into the lumen of one of the uterine horns (*b*). The contralateral uterine horn, which was not infused with oil, served as a nonstimulated control (*a*). Sections of the uterus were subjected to *in situ* hybridization using DIG-labeled LNA probes specific to *mir-320*. The scale bar indicated a distance of 100 μM. To evaluate the specificity of the probe, negative control staining was performed by substituting as DIG-labeled LNA-scrambled probe for the DIG-labeled LNA-miR-320 probe (*c*). Key: m, myometrium; m, maternal gland; s, stroma; le, luminal epithelium; db, decidua basalis.

To test whether *miR-320* expression was regulated by decidualization, a model of experimentally induced decidualization was used for Northern blot and *in situ* hybridization analyses. The expression level of *miR-320* in the decidualized uterus was evidently higher than in the nonstimulated uterus on day 7 of pseudopregnancy (Figure 4 A). *In situ* hybridization showed strong staining in glandular and luminal epithelia in the control uterine horn on day 7 of pseudopregnancy (Figure 4 B *a*). However, in the oil-infused uterus, strong signals were detected in decidua but staining was weak in the luminal epithelium (Figure 4 B *b*). This indicated that artificial decidualization promoted the expression of *miR-320* and further emphasize the importance of decidualization in regulating the dynamics of *miR-320* expression in the uterus during the window of implantation.

### 2.5. Progesterone enhances the miR-320 expression

Ovarian progesterone and estrogen are the principal hormones that direct uterine receptivity, embryo implantation and the maintenance of pregnancy in all mammals studied, and are essential for implantation in mice and rats [19,20].

**Figure 5.**
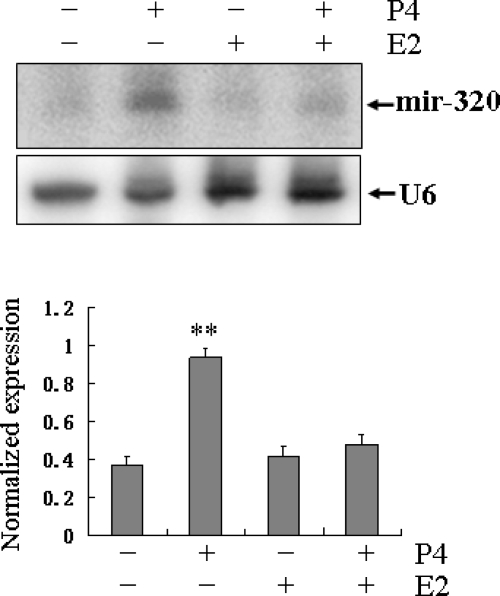
The effect of steroid hormones on uterine *miR-320* expression. The effect of steroid hormones on uterine *miR-320* expression steroid hormones on uterine *miR-320* expression was detected by Northern blot. Hybridization was done with a ^32^P-labeled probe for *miR-320* and *U6*. The black curve represents the optical densities of the signals quantified by densitometric analysis and represented as a ratio of *miR-320* to *U6* intensity to normalize for gel loading and transfer.

In order to test the effect of steroid hormones on the *miR-320* expression under physiological condition, Northern blot was performed to examine whether the *miR-320* expression was regulated by steroid hormones. A low level of *miR-320* expression was detected in the ovariectomized rat uterus. However, treatment with progesterone significantly increased *miR-320* expression (*P* < 0.01) and estradiol-17β did not visibly affect the expression level of *miR-320*. The *miR-320* expression was slightly increased by combination of both (Figure 5). All these facts indicated that progesterone can promote *miR-320* expression under physiological condition.

Progesterone (P_4_) is essential for the development of endometrial receptivity for blastocyst implantation under pregnant condition [28,29] and play an important role on the maintenance of female endocrine homeostasis by inhibiting the secretion of gonadotropin in hypothalamus. Endometrial receptivity for embryo implantation in the rat occurred on day 5 of pregnancy. The expression of *miR-320* is decreased on g.d.5, but its expression was increased by progesterone under physiological condition. These results implied that the low expression of *miR-320* may be benefit to formation of endometrial receptivity under pregnant condition and the high expression of *miR-320* may be in favor of the maintenance of female endocrine homeostasis. In addition, there are striking similarities between the behavior of invasive placental cells and that of invasive cancer cells. Dysregulated expression of the *miR-320* in many tumor [15,16] was strongly associated with tumor development, and *miR-320* expression level was down-regulated greater than two fold in primary breast cancer (BC) [15]. These results imply that the action of progesterone on *miR-320* expression might inhibit the excess invasion of cells from the uterus and trophectoderm.

## 3. Experimental Section

### 3.1. Experimental animals and protocols

Sexually mature, healthy female Sprague Dawley rats (220–260 g body weight) were purchased from the Laboratory Animal Center of the Academy of Military Medical Sciences (Beijing, PR China). The rats were housed in a temperature- and humidity-controlled room with a 12/12 h light/dark cycle. All animal procedures were approved by the Institutional Animals Care and Use Committee of the National Research Institute for Family Planning. The rats were caged overnight with fertile males of the same strain. The presence of a vaginal plug or sperm was considered to be day 1 of pregnancy (g.d.1). The whole uterus was collected from g.d. 3–5 rats. When embryos implant and placentae form, placentae were carefully peeled from dissected uterine horns and whole uterus including the peritoneum, myometrium and maternal decidua was collected from g.d. 6–9 rat. Divided uteri were respectively frozen in Eppendorf tubes and stored at 80 °C until processing for RNA extraction. Whole uteri or undivided uteri and placentae were fixed in 4% paraformaldehyde (PFA) solution (Sigma-Aldrich, St. Louis, MO, USA) in 0.1M phosphate buffer (pH7.4, 4 °C) for *in situ* hybridization analysis.

Pseudopregnancy was induced by caging adult females with vasectomized males, and mating was confirmed by checking for a vaginal plug (day 1 of pseudopregnancy). The whole uteri were collected from days 3–7 of pseudopregnancy. On day 5 of pseudopregnancy, when the uteri were optimally sensitized to deciduogenic stimuli, 100 μL olive oil (Sigma-Aldrich) was infused into the lumen of one of the uterine horns to induce artificial decidualization. The contralateral uterine horn, which was not infused with oil, served as a control. At day 7 of pseudopregnancy, the rats were sacrificed and the uterine horns were isolated.

To induce delayed implantation, the pregnant rats on g.d. 4 were ovariectomized. Progesterone (5 mg/rat, s.c.; Sigma-Aldrich) was injected to maintain delayed implantation from g.d. 5–7. The progesterone-primed delayed-implantation rats were treated with estradiol-17β (0.5 μg/rat; Sigma-Aldrich) to terminate delayed implantation. The rats were sacrificed by stunning and cervical dislocation to collect uteri 24 h after estrogen treatment. The implantation sites were also identified by i.v. injection of Chicago blue solution (Sigma-Aldrich). Delayed implantation was confirmed by flushing the blastocysts from the uterus.

To test the effects of steroid hormones on *miR-320* expression, rats were treated with hormones starting 2 weeks after they were ovariectomized. The ovariectomized rats were treated with an injection of estradiol-17β (1 μg/rat) or progesterone (10 mg/rat) at intervals of 24 h for 3 d. All steroids were dissolved in olive oil and injected subcutaneously. Controls received the vehicle only (0.1 mL/rat).

### 3.2. Northern blot analysis

Northern blot analysis of miRNAs was performed as described previously [22]. Briefly, total RNA was isolated from the uteri of rats with TRIzol reagent (Invitrogen, Carlsbad, CA, USA). Aliquots of 40 μg of total RNA per sample were subjected to electrophoresis on a 15% urea-PAGE gel and transferred to a nylon membrane (Hybond N+; Amersham Pharmacia Biotech, St Albans, Hertford, UK). After being UV cross-linked and baked at 50 °C for 30 min, the membrane was prehybridized at 42 °C for 4 h and then hybridized with ^32^P-labeled probes at 40 °C overnight. Membranes were washed and exposed to PhosphorImager screens (GE Healthcare Bio-Sciences Corp., Piscataway, NJ, USA). The bands were analyzed using the Quantity One software (Bio-Rad, Hercules, CA, USA). All experiments were repeated at least three times.

### 3.3. In situ hybridization of miR-320 with DIG-labeled LNA probes

*In situ* hybridization of miRNAs with DIG-labeled LNA probes was performed as described previously [23]. Briefly, sections of uterus (5 μm) were treated with proteinase K (20 g/mL) for 15 min and refixed in 4% PFA for 15 min. After acetylation with 0.25% acetic anhydride in 0.1 M triethanolamine (pH 8.0) for 10 min, sections were prehybridized with hybridization buffer (Roche, Mannheim, Germany) at 40 °C for 2h and then hybridized with digoxigenin (DIG)-labeled LNA-miR-320 probe (LNA-miR-320 sequence: 5′–DIG–ttCgcCctCtCaAcCcAgCtttt–3′) at 40 °C overnight. The cells were then incubated in buffer containing anti-DIG-antibody for 2 h at 37 °C and stained with 5-bromo-4-chloro-3-indolyl phosphate (BCIP; Promega, Madison, WI, USA) and p-nitroblue tetrazolium chloride (NBT; Promega, Madison, WI, USA). The cells and sections were hybridized with a DIG-labeled LNA-scrambled probe (LNA-scrambled sequences: 5′–caTtaAtgTcGgaCaaCtcAat–3′) as a negative control [24]. Samples were viewed with an Eclipse 80i microscope (Nikon, Tokyo, Japan).

### 3.4. Statistical analysis

There were at least three rats in each treatment group. The results of Northern blot and *in situ* hybridization were repeated three times. All values are reported as the mean ± SE. Statistical analysis was performed using one-way ANOVA. When significant effects of treatments were indicated, the Student–Newman–Keuls multiple range test was applied using SPSS version 13.0 (SPSS Inc., Chicago, IL, USA). *P <* 0.05 was considered statistically significant.

## 4. Conclusions

In conclusion, we found that the miRNA *miR-320* could be detected differentially in the rat uterus during the peri-implantation period. The results obtained from our models of pseudopregnancy, artificial decidualization and delayed implantation imply an important role for implanting blastocysts and decidualization in the temporal and spatial changes of *miR-320* expression in the uterus during the window of implantation. In addition, this expression of *miR-320* was regulated by progesterone. Collectively, these findings will help us gain a better understanding of the role of *mir-320* during pregnancy and provide a foundation for futher experimental studies on mechanisms mechanisms associated with the onset of uterine receptivity and embryo implantation.
